# Analysing Cooking Behaviour in Home Settings: Towards Health Monitoring †

**DOI:** 10.3390/s19030646

**Published:** 2019-02-04

**Authors:** Kristina Yordanova, Stefan Lüdtke, Samuel Whitehouse, Frank Krüger, Adeline Paiement, Majid Mirmehdi, Ian Craddock, Thomas Kirste

**Affiliations:** 1 Department of Computer Science, University of Rostock, 18051 Rostock, Germany; stefan.luedtke2@uni-rostock.de (S.L.); thomas.kirste@uni-rostock.de (T.K.); 2 Department of Electrical and Electronic Engineering, University of Bristol, Bristol BS8 1UB, UK; sw12690@bristol.ac.uk (S.W.); Ian.Craddock@bristol.ac.uk (I.C.); 3 Department of Computer Science, University of Bristol, Bristol BS8 1UB, UK; majid@cs.bris.ac.uk; 4 Department of Communications Engineering, University of Rostock, 18051 Rostock, Germany; frank.krueger@uni-rostock.de; 5 Department of Computer Science, University of Toulon, 83957 Toulon, France; adeline.paiement@univ-tln.fr

**Keywords:** activity recognition, plan recognition, goal recognition, behaviour monitoring, symbolic models, probabilistic models, sensor-based reasoning

## Abstract

Wellbeing is often affected by health-related conditions. Among them are nutrition-related health conditions, which can significantly decrease the quality of life. We envision a system that monitors the kitchen activities of patients and that based on the detected eating behaviour could provide clinicians with indicators for improving a patient’s health. To be successful, such system has to reason about the person’s actions and goals. To address this problem, we introduce a symbolic behaviour recognition approach, called Computational Causal Behaviour Models (CCBM). CCBM combines symbolic representation of person’s behaviour with probabilistic inference to reason about one’s actions, the type of meal being prepared, and its potential health impact. To evaluate the approach, we use a cooking dataset of unscripted kitchen activities, which contains data from various sensors in a real kitchen. The results show that the approach is able to reason about the person’s cooking actions. It is also able to recognise the goal in terms of type of prepared meal and whether it is healthy. Furthermore, we compare CCBM to state-of-the-art approaches such as Hidden Markov Models (HMM) and decision trees (DT). The results show that our approach performs comparable to the HMM and DT when used for activity recognition. It outperformed the HMM for goal recognition of the type of meal with median accuracy of 1 compared to median accuracy of 0.12 when applying the HMM. Our approach also outperformed the HMM for recognising whether a meal is healthy with a median accuracy of 1 compared to median accuracy of 0.5 with the HMM.

## 1. Introduction and Motivation

One aspect of having a healthy lifespan is the type and way in which we consume food [1]. Following an unhealthy diet can cause nutrition-related diseases, which in turn can reduce the quality of life. This is especially observed in prolonged physical conditions, such as diabetes and obesity, or mental health conditions such as eating disorders and depression. Such conditions influence one’s desire to prepare and consume healthy meals, or, in some cases, the patient’s ability to prepare food, e.g., those suffering from dementia disorders whose abilities are affected by the disease’s progression [2]. Such conditions are also associated with high hospitalisation and treatment costs. Different works have attempted to solve this problem by providing automated home monitoring of the patient. Potentially, this can also improve the well-being of the patient by replacing hospitalisation with monitoring and treatment in home settings [3,4].

A system able to address the above problem has to recognise one’s actions, goals and causes of the observed behaviours [5]. To that end, different works propose the application of knowledge-based models often realised in the form of ontologies [6,7,8]. In difference to data-driven methods, which need large amounts of training data and are only able to learn cases similar to those in the data, knowledge-based approaches can reason beyond the observations due to their underlying symbolic structure. This symbolic representation defines all possible behaviours and the associated effects on the environment. In that manner, they can reason about the one’s actions, goals, and current situation [2]. While rule-based approaches provide additional unobserved information, they have two main disadvantages when modelling problems in unscripted settings: (a) behaviour complexity and variability results in large models that, depending on the size, could be computationally infeasible; and (b) noise typical for physical sensors results in the inability of symbolic models reason about the observed behaviour.

In attempt to cope with these challenges, there are works that propose the combination of symbolic structure and probabilistic inference (e.g., [9,10,11]). This type of approaches are known, among other, as Computational State Space Models (CSSMs) [12]. These approaches have a hybrid structure consisting of symbolic representation of the possible behaviours and probabilistic semantics that allow coping with behaviour variability and sensor noise [9,11,12]. Currently, CSSMs have shown promising results in scripted scenarios but have not been applied in real world settings. In other words, the models have been applied on simplified problems that do not extensively address the complications caused by behaviour complexity and variability observed in real settings. Another core challenge is the recognition of one’s high-level behaviour from low level sensor observations [13].

In a previous work, we shortly presented our concept and we showed first preliminary empirical results from CSSMs applied to an unscripted scenario [14]. In this work, we extend our previous work by providing

1. detailed formal description of the proposed methodology;
2. detailed analysis on the influence of different sensors on the CSSM’s performance;
3. extension of the model to allow the recognition of single pooled and multiple goals;
4. detailed comparison between state-of-the-art methods and our proposed approach.

The work is structured as follows. Section 2 discusses the state of the art in symbolic models for activity and goal recognition. Section 3 presents the idea behind CSSMs and discusses a concrete implementation of CSSMs, called Computational Causal Behaviour Models, which we used in this work. Section 4 describes the experimental setup and the model development while Section 5 presents the results. The work concludes with a discussion and outline of future work in Section 6 and Section 7, respectively.

## 2. Related Work

To be able to reason about one’s cooking and eating behaviour and its implications on their health, a system has to be able to infer information about the person’s behaviour from observation by means of sensors. To this end, current work distinguishes among Activity Recognition (AR), goal recognition (GR) and Plan Recognition (PR) [13]. AR is known as the task of inferring the user’s current action from noisy and ambiguous sensor data, while GR deals with recognising the goal the person is pursuing. Plan recognition, in contrast, aims to infer the action sequence leading to a goal under question by using (partial) action observations. Plan recognition can be considered as the combination of activity and goal recognition. In other words, PR recognises both the person’s sequence of actions and goals they follow.

The term “behaviour recognition”, on the other hand, refers to the overall process of activity, goal, and plan recognition [15]. In the framework of behaviour recognition, we refer to “actions” as the primitive actions constituting a plan. A plan is then a sequence of actions leading to a certain goal. Note that there is some variation in the interpretation of the term “activity recognition” across different works. For example, the authors of [8] referred to activity recognition as the process of recognising coarse-grained complex activities (e.g., “prepare breakfast”, “take a shower”, etc.) They however did not recognise the sequence of fine-grained actions needed to complete these activities. In our work, we consider the recognition of these fine-grained actions as “activity recognition”, the recognition of the correct sequence of fine-grained actions as “plan recognition”, and the recognition of the overall complex activity as “goal recognition”. In difference to typical AR terminology, where actions can build up complex parallel or interleaving activities, here we consider plan to be complex activity built up of sequentially executed actions. Based on this definition, we introduce the general model of sensor based behaviour recognition which has the objective to label temporal segments by use of observation data.

**Figure 1:**
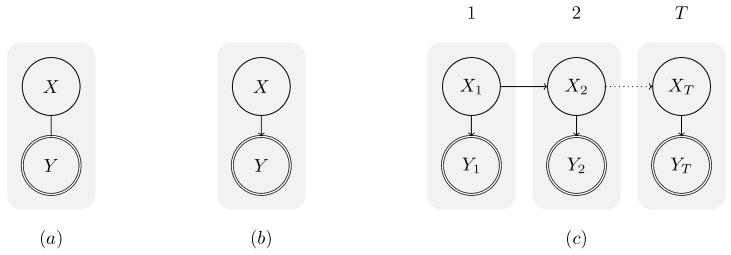
Graphical representation of three different types of classifier, where *X* represents a hidden state and *Y* an observation that is used to conclude information about *X*: (**a**) discriminative classifier; (**b**) generative classifier without temporal knowledge; and (**c**) generative classifier with temporal knowledge (figure adapted from [16]).

From the inference point of view, the aim of behaviour recognition is to estimate a hidden variable *X* from an observable variable *Y*. Figure 1a provides a graphical illustration of this task. Here, the hidden variable *X* represents the activity of the human protagonist.

With respect to the probabilistic structure, two fundamentally different approaches to behaviour recognition exist [17]: discriminative and generative classifiers. While discriminative classifiers model the conditional probability P(X|Y), generative classifiers model the joint probability P(X,Y). In other words, discriminative classifiers map the observation data to activity labels directly [17], while generative classifiers allow exploiting the causal link between the system’s state *X* and the observation *Y* by factoring the joint probability into P(X,Y)=P(Y|X)P(X). A graphical representation of generative models is provided in Figure 1b. The representation allows including prior knowledge about the dynamics of the underlying process. Furthermore, experiences can often be used to establish the sensor model P(Y|X) [18] (p. 5). Bulling et al. [19] provided a detailed overview of methods for activity recognition. Typical approaches include decision trees [20], support vector machines [21,22], or random forests [23,24].

By including further knowledge about the temporal structure of the activities, a transition model $P(X_{t}|X_{t-1})$ can be introduced to provide temporally smoothed estimates of the activities. This is illustrated in Figure 1c. Generally, temporal generative models (e.g., Hidden Markov Models (HMM)) do not raise any restrictions to the possible sequences of activities, which allows the estimation of sequences that are impossible from the viewpoint of causality.Then, to reason about the causally valid action sequences, we need to use plan recognition (PR) approaches. The literature provides different overviews of plan recognition [13,25,26,27,28].

From the modelling viewpoint, two different approaches exist for restricting the set of possible sequences. The first approach is to enumerate all possible sequences (plans) in a plan library [29]. However, the plan library has to be created manually, which is a tedious task due to the high number of action sequences [30]. As stated in [11], “library-based models are inherently unable to solve the problem of library completeness caused by the inability of a designer to model all possible execution sequences leading to the goal”. This is especially true in real world problems where the valid behaviour variability can result in models with millions of states and execution sequences. Note that by “valid behaviour” we mean any causally correct sequence of actions. This does not necessarily mean that the behaviour is also rational from a human point of view. “Causally correct” here indicates that the sequence of actions is physically possible (e.g., a person has to be at a given location to execute the action at this location, or an object has to be present for the person to interact with this object, etc.

A second approach to introducing restrictions to the set of causally valid action sequences is to employ a structured state representation of *X* and generate only action sequences that are causally valid with respect to this state. This technique is also known as inverse planning [31], as it employs ideas from the domain of automated planning to infer the action sequence of a human protagonist. This technique was, for instance, used by Geib and Goldman [32] and Ramírez and Geffner [10].

To cope with behaviour variability, some works introduce the use of computational state space models (CSSM). CSSMs allow modelling complex behaviour with a lot of variability without the need of large training datasets or manual definition of all plans [9,10]. CSSMs describe the behaviour in terms of precondition-effect rules and a structured state representation to synthesise possible action sequences. The manually implemented model is very small as it requires only a few rules to generate different sequences of possible actions. This is done through the causal relations defined in the rules. Note that this is an alternative to manually defining all possible action sequences, or using large training datasets to learn them. In addition, some CSSMs combine their symbolic structure with probabilistic inference engines allowing them to reason even in the presence of ambiguous or noisy observations [11,12].

Table 1 lists different works on CSSMs.

For example, most of them make simplifying assumptions of the environment to perform plan recognition. Most do not make use of action durations. In the real world, however, actions have durations and this makes the inference problem more complex. Many works assume perfect observations, which is not the case in real world problems where the sensors are typically noisy and there are missing observations. In addition, most works assume that there is only one goal being followed. In real world scenarios, it is possible that the goals change over time. Presently, CSSMs have been applied to problems in scripted scenarios. Such settings limit the ability to investigate behaviour complexity and variability that is usually observed in everyday life. There is still no solid empirical evidence that CSSMs can cope with the behaviour variability observed in real world everyday activities (note that CSSMs have been applied to the problem of reconstructing the daily activities in home settings [37]. This analysis, however, is very coarse-grained and does not address the problem of reasoning about the fine-grained activities and goals within a certain task, e.g., while preparing a meal). Additionally, presently, CSSMs have been used for recognising the goal only in scenarios with simulated data [9,10]. In other words, it is unclear whether these approaches are able to reason about the user behaviour when the user is not following a predefined script or agenda.

In a previous work, we showed preliminary results of a CSSM approach called Computational Causal Behaviour Models (CCBM), which reasons about one’s activities in real cooking scenarios [14,38]. In this work, we extend our previous work by providing detailed information on the approach and the developed model. We show that our model is able to perform goal recognition based on both multiple and single “pooled” goals. We also present a detailed empirical analysis on the model performance, the effect of the sensors on the performance, and we compare the proposed approach with state-of-the-art Hidden Markov Model for PR. Finally, we provide a discussion on the effect of adding the person’s position extracted from video data on the model performance.

## 3. Computational Causal Behaviour Models

**Figure 2:**
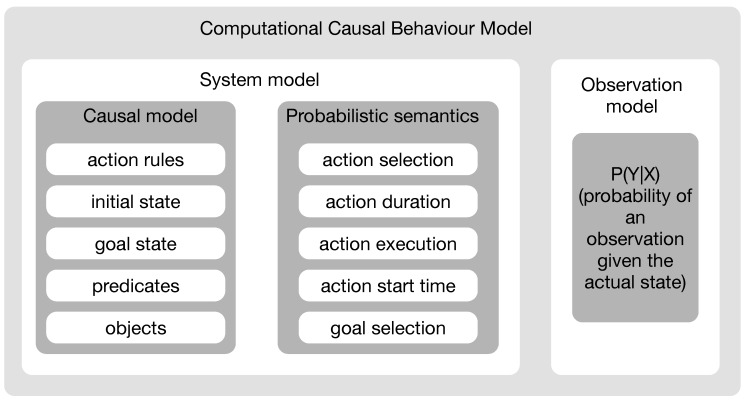
Elements of a Computational Causal Behaviour Model (CCBM).

The previous section introduces the concept of behaviour recognition and illustrates that CSSMs provide a convenient approach by bridging the gap between activity recognition and plan recognition. This section further extends these concepts and gives an introduction to Computational Causal Behaviour Models, a framework for sensor based behaviour recognition based on the principle of CSSMs. The description is based on the dissertation of Krüger [16]. Figure 2 describes the CCBM elements.

CCBM provides a convenient way to describe sequences of human actions by means of computable functions rather than by providing plan libraries to provide all possible sequences. From the viewpoint of probabilistic models, as introduced in Section 2, CCBM allows specifying the temporal knowledge of human behaviour—the system model $P(X_{t}|X_{t-1})$. To this end, CCBM employs a causal model, which uses precondition and effect rules in order to describe the system dynamics. Besides the system model, inference from noisy observations requires an observation model, which basically describes the relation between the sensor observation *Y* and the system’s state *X*. CCBM uses a probabilistic semantic to cope with uncertainties resulting from noisy sensor observations and the non-deterministic human behaviour. In the following, these concepts are described in more detail.

### 3.1. Causal Model

**Figure 3:**
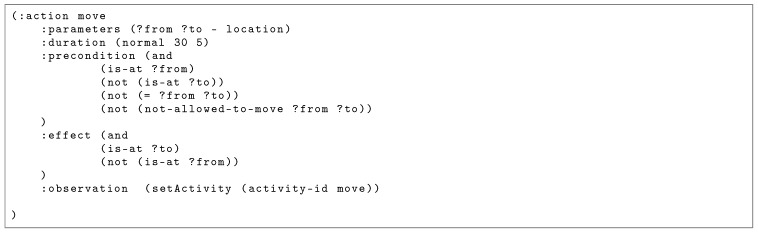
Example rule for the execution of the action “move”.

As described above, CSSMs rely on the idea of plan synthesis. A model-based description [39] is employed to describe the state space and possible actions. A state is described by predicates, where each represents a property of the environment such as the current position of the person, or whether the person is hungry. Actions are described by means of preconditions and effects with respect to a structured state. While the preconditions restrict the application of actions to appropriate states, the effects describe how the state is changed after executing an action. In the case of CCBM, the causal model employs a Planning Domain Definition Language (PDDL)-like [40] notation to describe the action rules, the initial and goal states, and the concrete objects in the environment. Figure 3 shows an example of a PDDL-like rule for the action “move”.

The rule represents an action template that can be grounded with different parameters. For example, the action template “move” can be grounded with two parameters of type “location” (e.g., “(move ?from ?to)” could yield the concrete action “(move kitchen study)”). The template then incorporates its rules in terms of preconditions and effects. The preconditions describe the constraints on the world in order for an action to become executable, while the effects describe how the execution of this action changes the world. They are defined in terms of predicates that describe properties of the world. Apart from the preconditions and effects, an action template has a duration (in this case, a normal distribution with a mean of 30 and a standard deviation of 5), and an “:observation” clause that maps the high level action to the observation model. Apart from the action templates, the objects in the environment and their types are expressed in a PDDL-like notation.

**Figure 4:**
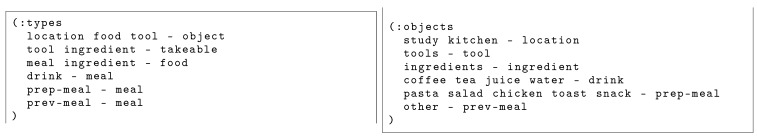
Example definition of types and their concrete objects for the cooking problem.

Figure 4 shows the definition of the types and objects for the cooking problem. For example, the objects “study” and “kitchen” are from type “location” and the type “location” has a parent type “object”. This notation builds up a hierarchy with the concrete objects at the bottom and the most abstracted class at the top. A graphical representation for this structure can be seen in Figure 7.

The action templates together with the objects generate a symbolic model that consists of actions A, states S, ground predicates P, and plans B. The set A of grounded actions is generated from the action templates by grounding a template with all possible concrete objects. The set P of grounded predicates is generated by grounding all predicates with the set of all possible objects. A state s∈S is defined as a combination of all ground predicates and describes one particular state of the world. For a state s∈S, each state describes one possible combination of the actual values of the ground predicates. Imagine we have a model that has three predicates and each of these predicates can have the value true or false. One possible state would have the following representation $s=(pr_{1}:=\mathrm{true},pr_{2}:=\mathrm{true},pr_{3}:=\mathrm{false})$. Then, S represents the set of all such predicate combinations, i.e., all states that are reachable by applying any sequence of action on the initial state. We can reach from one state *s* to another $s^{\prime }$ by applying an action a∈A in *s*. We then say that $s^{\prime }$ is reachable from *s* by *a*. The initial state $s_{0}$ is the state of the world at the beginning of the problem. Apart from the initial state, another important subset of states is the one containing the goal states G⊆S. G is basically the set of states containing all predicates that have to be true in order to reach the goal the person is following. $B=\{p_{1},\ldots ,p_{n}\}$ is the set of all possible execution sequences (or plans) starting in the initial state and reaching a goal state. For a plan $p_{i}=\left(a_{1},\ldots ,a_{m}\right)$ that leads to a goal g⊆G and an initial state $s_{0}\in S$, we say that $p_{i}$ *achieves* G, if for a finite sequence of actions $a_{j}$ and a state $s_{l}\in G$, $s_{l}=a_{m}\left(\cdots a_{2}\left(a_{1}\left(s_{0}\right)\right)\cdots \right)$. B corresponds to a plan library in approaches that manually define the possible plans [29]. A plan library will then contain all possible valid plans (either manually built in library-based approaches or generated from rules in approaches such as CCBM).

### 3.2. Probabilistic Semantics

**Figure 5:**
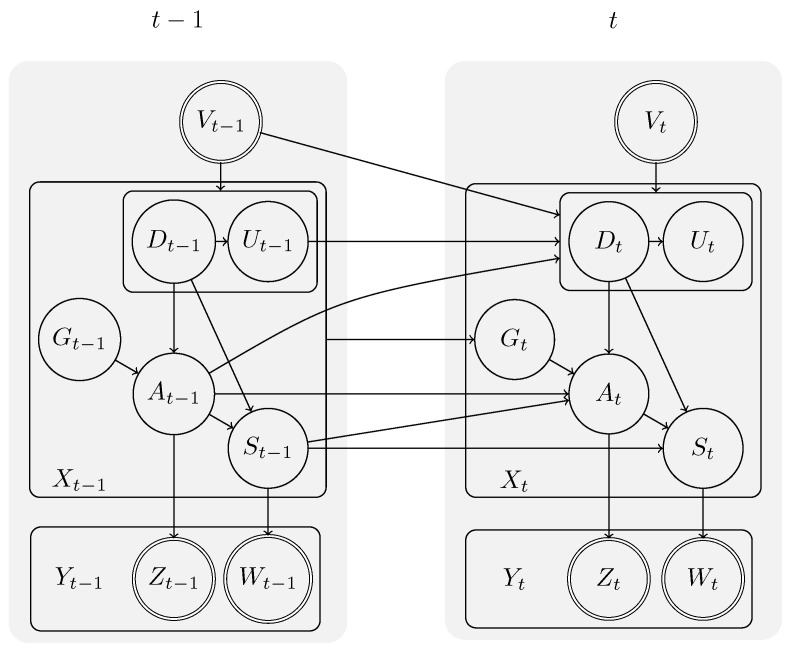
DBN structure of a CCBM model. Adapted from [12].

As introduced in Section 2, a generative probabilistic model with temporal knowledge is modelled by two random variables *X* and *Y* at different time steps, which represent the belief about the system’s state and the sensor observation. To reason not only about the state, but also about the action being executed by the person, the state *X* is further structured. As can be seen in Figure 5, the system state *X* is structured into the following random variables S,A,G,D, and *U*, each representing a different aspect of the state. Similarly, the observation *Y* is structured into Z,W, and *V*, each representing different types of sensory observation.

From the illustration, it can be seen that the state *X* at time *t* depends on the state at time t−1. The transition model describes this dependency through the probability $P(X_{t}|X_{t-1})$ of observing that we are in a state *X* given that the the previous state was $X_{t-1}$. We call this model a **system model**. The state *X* is a five-tuple (A,D,G,S,U), which can be rewritten as:

(1) $$\begin{array}{c} p\left(X_{t}|X_{t-1}\right)=p\left(A_{t},D_{t},G_{t},S_{t},U_{t}|A_{t-1},D_{t-1},G_{t-1},S_{t-1},U_{t-1},V_{t},V_{t-1}\right) \end{array}$$

where the random variable *A* represents the action (from the set A) that is currently executed by the human protagonist while trying to achieve the goal *G* (from the set G). The current state of the environment is represented by *S* (from the set S). The variables *U* and *D* represent the starting time of the current action and signal whether the action should be terminated in the next time step. Finally, the variables *V*, *W*, and *Z* reflect observations of the current time, environment, and action, respectively.

By exploiting the dependencies from the graphical model, this transition model can be simplified into five sub-models, each describing the dependency of one element of the state.

(2) $$\begin{array}{ccc} p(S_{t}|A_{t},D_{t},S_{t-1}) & ▸ & \;\mathrm{action}\;\mathrm{execution}\;\mathrm{model} \end{array}$$

(3) $$\begin{array}{ccc} p(A_{t}|D_{t},G_{t},A_{t-1},S_{t-1}) & ▸ & \;\mathrm{action}\;\mathrm{selection}\;\mathrm{model} \end{array}$$

(4) $$\begin{array}{ccc} p(U_{t}|D_{t},U_{t-1},V_{t}) & ▸ & \;\mathrm{action}\;\mathrm{start}\;\mathrm{time}\;\mathrm{model} \end{array}$$

(5) $$\begin{array}{ccc} p(D_{t}|A_{t-1},U_{t-1},V_{t},V_{t-1}) & \;▸ & \;\mathrm{action}\;\mathrm{duration}\;\mathrm{model} \end{array}$$

(6) $$\begin{array}{ccc} p(G_{t}|X_{t-1}) & ▸ & \;\mathrm{goal}\;\mathrm{selection}\;\mathrm{model} \end{array}$$

The **action execution model** describes the application of the actions to a state and the resulting state. This sub-model basically implements the effects of the action description. For this purpose, the resulting state is determined based on the current state, the action to be executed, and whether the current action is finished. More formally,

(7) $$\begin{array}{c} p\left(s_{t}|a_{t},d_{t},s_{t-1}\right)=\left\{\begin{array}{cc} 1, & \mathrm{if}\;d_{t}=\mathrm{false}\wedge s_{t}=s_{t-1};\\ 1, & \mathrm{if}\;d_{t}=\mathrm{true}\wedge s_{t}=a_{t}\left(s_{t-1}\right);\\ 0, & \mathrm{otherwise}. \end{array}\right. \end{array}$$

Depending on the value of $d_{t}$, either the system’s state remains untouched, or the new state $s_{t}$ is set to the results of applying the selected action $a_{t}$ to the current state $s_{t-1}$. While the action execution model generally allows the usage of non-deterministic effects (i.e., the outcome of an action is determined probabilistically), CCBM’s deterministic effects are considered sufficient in order to model the human protagonist’s knowledge about the environment.

Both the **action start time** and the **action duration model** implement the fact that actions executed by human protagonists consume time. While the first model basically “stores” the starting time of the current action, the second model determines whether the action that is currently executed should be terminated or not. From the probabilistic viewpoint, the action start time model is rather simple. The value of $u_{t}$ is set to the current time *t* if a new action is selected, otherwise the value is copied from the previous time step. More formally,

(8) $$\begin{array}{c} p\left(u_{t}|d_{t},u_{t-1},v_{t}\right)=\left\{\begin{array}{cc} 1, & \mathrm{if}\;\left(d_{t}=\mathrm{false}\wedge u_{t}=u_{t-1}\right)\vee \left(d_{t}=\mathrm{true}\wedge u_{t}=v_{t}\right);\\ 0, & \mathrm{if}\;\left(d_{t}=\mathrm{false}\wedge u_{t}\neq u_{t-1}\right)\vee \left(d_{t}=\mathrm{true}\wedge u_{t}\neq v_{t}\right). \end{array}\right. \end{array}$$

The action duration model employs the start time of an action to determine the termination by use of an action specific duration function (provided by the CDF *F*):

(9) $$\begin{array}{c} p\left(d_{t}|a_{t-1},u_{t-1},v_{t},v_{t-1}\right)=\frac{F\left(v_{t}|a_{t-1},u_{t-1}\right)-F\left(v_{t-1}|a_{t-1},u_{t-1}\right)}{1-F(v_{t}|a_{t-1},u_{t-1})} \end{array}$$

The objective of the **goal selection model** is to provide a mechanism to model the rational behaviour of the human protagonist. While different approaches in the literature allow changes of the goal (see, for instance, [31,41]), CCBM is based on the assumption that, once a goal is selected, it is not changed. Similar as for the deterministic action execution, this assumption is based on the complete and static knowledge of the human protagonist. This means that the goal is chosen at time t=0 and copied from the previous time step afterwards.

(10) $$\begin{array}{c} p\left(g_{t}|g_{t-1},s_{t-1},a_{t-1},d_{t-1},u_{t-1}\right)=\left\{\begin{array}{cc} 1, & \mathrm{if}\;g_{t}=g_{t-1}\\ 0, & \mathrm{otherwise} \end{array}\right. \end{array}$$

To reflect the human protagonist’s freedom to choose an action, the **action selection model** provides a probability distribution over possible actions. This includes two aspects:

1. goal-directed actions are preferred, but
2. deviations from the “optimal” action sequence are possible.

Whenever the previous action has been terminated, a new action has to be selected.

(11) $$\begin{array}{c} p\left(a_{t}|d_{t},g_{t},a_{t-1},s_{t-1}\right)=\left\{\begin{array}{cc} \gamma (a_{t}|g_{t},a_{t-1},s_{t-1}), & \mathrm{if}\;d_{t}=true\\ 0, & \mathrm{if}\;d_{t}=false\wedge a_{t}\neq a_{t-1}\\ 1, & \mathrm{if}\;d_{t}=false\wedge a_{t}=a_{t-1} \end{array}\right. \end{array}$$

This is done by the action selection function γ, which is implemented based on log-linear models. This allows including different factors into the action selection mechanism.

(12) $$\begin{array}{cc} \tilde{\gamma }\left(a_{t}|g_{t},a_{t-1},s_{t-1}\right)= & \exp\left(\sum_{k\in K}\lambda_{k}f_{k}\left(a_{t},g_{t},a_{t-1},s_{t-1}\right)\right) \end{array}$$

(13) $$\begin{array}{cc} \gamma (a_{t}|g_{t},a_{t-1},s_{t-1})= & \frac{1}{Z}\tilde{\gamma }\left(a_{t}|g_{t},a_{t-1},s_{t-1}\right) \end{array}$$

(14) $$\begin{array}{cc} Z= & \sum_{a\in A}\tilde{\gamma }\left(a_{t}|g_{t},a_{t-1},s_{t-1}\right) \end{array}$$

where $f_{k}$ is the feature used for the action selection with k={1,2}. $f_{1}$ is based on the goal distance [10], while $f_{1}$ uses a landmarks-based approximation of the goal distance [42]. The distance in $f_{1}$ is determined by an exhaustive process, which could be infeasible in very large state spaces. For that reason, $f_{2}$ uses approximation based on the predicates that have to be satisfied to reach the goal. The weight $\lambda_{k}$ allows the adjust the influence of the particular heuristic to the action selection model.

### 3.3. Observation Model

The connection between the system model and the observations is provided through the **observation model**. It gives the probability $p(Y_{t}=y_{t}|X_{t}=x)$ of observing an observation *y* given a state *x*. As illustrated in Figure 5, the random variable *Y* is structured into Z,W, and *V*, representing sensors observation of actions (e.g., movement of a person), the environment (e.g., whether a cupboard is open or closed) and the current time. While the observation model itself is not part of the causal model, but has to be provided separately, the :observation clause in the action template is used to map the specific functions from the observation model to the high level system model (see Figure 3). The :observation provides the observation model with information about the current state *X*, such as the action currently executed or the environment state. The objective of the observation model is to provide the probability of the sensor observation given the action a∈A and state s∈S.

(15) $$\begin{array}{c} P\left(Y_{t}|X_{t}\right)=P\left(Z_{t}|A_{t}\right)P\left(W_{t}|S_{t}\right) \end{array}$$

While there are no restrictions to the way in which this probability is computed, here we use an observation model that incorporates a decision tree. More information about the implementation of the system and observation models can be found in [43].

## 4. Experimental Setup and Model Development

### 4.1. Data Collection

To investigate whether CSSMs are applicable to real word scenarios, we used a sensor dataset recorded in real settings. The dataset consists of 15 runs of food preparation and eating activities. The dataset was collected in a real house rented by the SPHERE (a Sensor Platform for HEalthcare in a Residential Environment) project [44].

**Figure 6:**
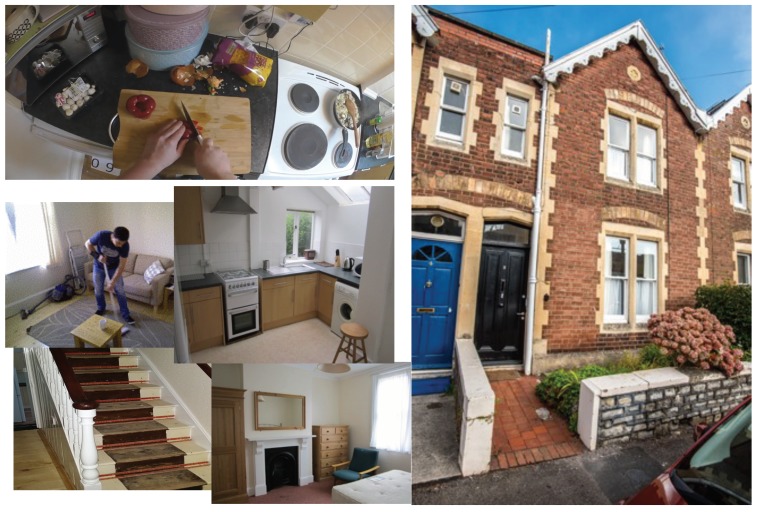
The SPHERE house at the University of Bristol and the kitchen setup.

SPHERE is an interdisciplinary research project aiming to assist medical professionals in caring for their patients by providing a monitoring solution in home settings [44]. The SPHERE House is a normal two-bedroom terrace in Bristol (UK), into which has been installed the full SPHERE system for testing, development and data collection. The SPHERE system consists of a house wide sensor network that gathers a range of environmental data (such as temperature, humidity, luminosity, and motion), usage levels (water and electricity), and RGB-D features from depth cameras in the downstairs rooms [45]. The system also has support for a wearable sensor, used for location, heart-rate and an on-board accelerometer, although this was not used for this dataset due to hygiene concerns. For this work, binary cupboard door state monitoring was also added in the house kitchen to provide additional domain relevant data [46]. A head-mounted camera was used during the data collection to provide a point-of-view for each participant allowing for easier annotation of the observations. Due to extensive consultation with members of the public and medical professionals, the SPHERE system strikes a balance between protecting the privacy of the occupants and providing useful clinical data. Figure 6 shows the SPHERE house and the kitchen setup.

Each participant was given ingredients that they had requested and asked to perform two or three cooking activities of their choice without any set recipe or other restrictions. They were encouraged to act naturally during the recordings, which lead to a range of behaviours, for example leaving the kitchen for various reasons, preparing drinks as desired and using personal electrical devices when not actively cooking. This led to various meals, in terms of both preparation time and complexity, and a range of exhibited behaviours. Table 2 shows the different runs, the meals and drinks that were prepared and whether they have been classified as healthy for the purposes of this work. It also shows the length of the task in terms of time steps after the data was preprocessed (see Section 4.2).

The resulting dataset can be downloaded from [47]. Note that this dataset does not contain the RGB-D data from the depth cameras as this was not stored by the system for privacy reasons. Bounding boxes of humans in the scene, generated by the system from the RGB-D in real time, are included, and were used in this work to evaluate whether the information from the cameras improves model performance (see Section 5.2).

### 4.2. Data Processing

The dataset was recorded in JSON format. After converting it into column per sensor format, the procedure generated multiple rows with the same timestamp (in milliseconds). To remove redundant data, we combined rows with the same timestamp given that there was only one unique value for a sensor type. Furthermore, the action class was added for each row in the data. As the conversion generates some undefined values (due to the different frequencies of data collection for each type of sensor), time steps with undefined values for a particular sensor were replaced with the nearest previous value for that sensor. Apart from collecting the sensors’ values at a certain sampling rate, the value was also recorded when a change in the sensor’s state was detected. This ensured that replacing undefined values with the previous one would not result in transferring incorrect values when the sensor changes its state. To reduce the impact of ambiguous observations on the model performance, we applied a sliding window for all sensors with overlap of 50%. We used a window size of 5. We summarised the values in a window by taking the maximum for each sensor. A larger window resulted in removing certain actions from the dataset. For that reason, we chose the window size of five steps. This still produced actions with equivalent observations but reduced their number. The length of the resulting execution sequences in time steps can be seen in Table 2.

### 4.3. Data Annotation

To obtain the annotation, an action schema and ontology were developed and used for the annotation and the CCBM model design considerations. The ontology contains all elements in the environment that are relevant for the annotation and the model, while the action schema gives the restrictions on which actions can be applied to which elements in the environment. Note that the proposed action schema is more fine-grained than other existing works on AR in home settings (e.g., see [37,48]). This could be partially explained with the fact that sensors are unable to capture fine-grained activities leading to the development of action schema on the sensors’ granularity level [49]. In this work, we follow the assumption that we need an action schema on the granularity level of the application domain, so we are not guided by the sensors’ granularity. In other words, we can produce annotation that is not captured by the sensors. To produce the annotation, we followed the process proposed in [50,51].

#### 4.3.1. Ontology

The ontology represents the objects, actions, and their relations that are relevant for the problem. The actions in the ontology refer to the meal they are contributing to (e.g., “get ingredient pasta” means “get the ingredient needed for preparing pasta”). The action schema used in the model are shown in Table 3.

They provide the rules for applying actions on elements in the environment. For example, *prepare* can be applied only on element of type *meal*. In Table 3, *location* and *item* represent sets of objects, necessary for achieving the task, while *meal* refers to the goal of preparing a certain type of meal. The concrete locations, items and meals can be seen in Table 4.

Here, the *get*, *put* and *prepare* actions do not take into account the location where the meal is being prepared but rather the type of meal as a goal. Besides the goal oriented actions, the ontology also has an *unknown* action, which describes actions that do not contribute to reaching the goal.

**Figure 7:**
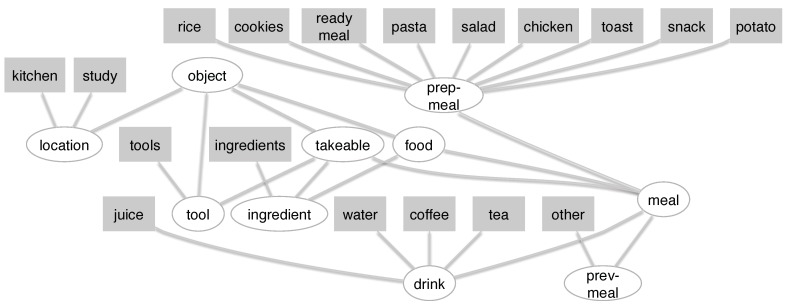
The relevant elements in the environment represented as hierarchy. Rectangles show objects; ellipses describe the object types; and arrows indicate the hierarchy or “is-a” relation (the arrow points to the father class). Figure adapted from [38].

Figure 7 shows the objects’ hierarchy for the action schema. The rectangles show the object used in the experiment, while the ellipses depict the object types. The types are the same as those in Table 4.

#### 4.3.2. Annotation

Based on the ontology, 15 datasets were annotated using the video logs from the head mounted camera and the ELAN annotation tool [52]. The process proposed in [50] was used, ensuring that the resulting annotation is syntactically and semantically correct.

Table 5 shows an example of the annotation, where the time indicates the start of the action in milliseconds. The annotation has then been mapped to the processed sensor data, where the time is no longer in milliseconds but in time steps.

The length of the annotation sequences after synchronising with the sensor data (with and without timing) can be seen in Table 2. The types of meals are also listed there. The annotation was later used to simulate data for the experiments reported in [38]. It was also used as a ground truth in this work during the model evaluation as well as for training the observation model for the CCBM.

### 4.4. Model Development

To develop the model, we followed the process proposed in [11]. It consists of developing the causal model and the observation model, then defining the different heuristics for action selection and finally identifying appropriate action durations.

#### 4.4.1. CCBM Models

##### Causal Model

We first developed 15 specialised models, which were fitted for each of the 15 execution sequences (we call these models $CCBM_{s}$). More specifically, for all 15 models, the same precondition-effect rules were used, but the initial and goal states differed so that they could accommodate the specific for each meal situation. We built the specialised models only for comparison purpose, as we wanted to evaluate whether our general model performs comparable to specialised models, which are typically manually developed in approaches relying on plan libraries. We also developed a general model, able to reason about all sequences in the dataset and allow performing GR (we call this model $CCBM_{g}$). The model uses the action schema presented in Figure 3 and can label the observed actions as one of the following classes: *clean*, *drink*, *eat*, *get*, *move*, *prepare*, *put*, and *unknown*.

The model size in terms of action classes, ground actions, states, and plans for the different model implementations can be seen in Table 6. Here, “action classes” shows the number of action types in the model, “ground actions” gives the number of unique action combinations based on the action schema in Table 3, “states” gives the number of S-states the model has, and “valid plans” provides the number of all valid execution sequences leading from the initial to one of the goal states. The general model has a larger state space and a larger set of plans. This allows coping with behaviour variability, however, it potentially reduces the model performance in contrast to the over-fitted specialised model.

##### Goals in the Model

To test whether the approach is able to reason about the observed behaviour, we modelled the different type of behaviour as different goals. As we were interested not only in the type of meal the person is preparing, but also what influence it has on the person’s health, we integrated two types of goals:

1. Type of meal being prepared: Here, we have 13 goals, which represent the different meals and drinks the person can prepare (see Table 4).
2. healthy/unhealthy meal/drink (4 goals): Here, we made the assumption that coffee, toast, and ready meals are unhealthy, while tea and freshly prepared meals are healthy (see Table 2). This assumption was made based on brainstorming with domain experts.

Note that, although the goals could also be interpreted as actions or states in difference to AR approaches, in GR, we are able to predict them before they actually happen.

##### Duration Model

The action durations were calculated from the annotation. Each action class received probability that was empirically calculated from the data. The probability models the duration of staying in the one state.

**Figure 8:**
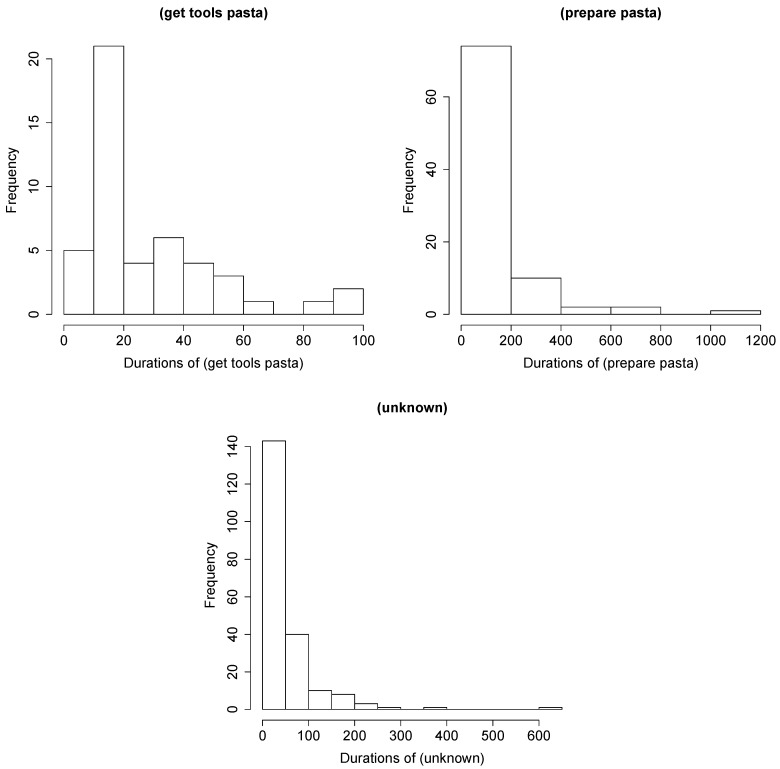
Frequency of the durations of some actions in the dataset.

Figure 8 shows selected examples of the frequency of action durations for a given action.

##### Observation Model

The observation model P(y|x) has been obtained by a learning-based approach as follows. We trained a decision tree (DT) based on the action classes. The output of the DT is for each time step, a distribution of the action class, given the current observation. This output has been used as a high-level observation sequence that is used to update the state prediction of the CCBM, by weighting each state by the probability of the corresponding action class, as indicated by the DT. The DT was applied to the following sensors:

- *Fridge electricity consumption:* For this sensor, we expected to see more electricity consumption when the door of the fridge is open, which would indicate an ingredient being taken from the fridge.
- *Kitchen cupboard sensors (top left, top right, sink)* show whether a cupboard door is open, which could indicate that an ingredient or a tool has been taken from the cupboard;.
- *Kitchen drawer sensors (middle, bottom):* As with the cupboard sensors, they provide information on whether a drawer has been opened.
- *temperature sensor* measures the room temperature and increasing temperature can potentially indicate the oven or stoves being used.
- *Humidity sensor* measures the room humidity and increased humidity can indicate cooking (especially when boiling water for the pasta).
- *Movement sensor* provides information whether a person is moving in the room. This is useful especially for the eating actions, when the person leaves the kitchen and eats in the study.
- *Water consumption (hot and cold)* shows the water consumption in the kitchen. This is useful especially in the cleaning phase.
- *Kettle electricity consumption:* As with the fridge, we expected to see more electricity consumption when the kettle is on and somebody is boiling water.
- *Depth cameras:* The position of the person was estimated through the depth cameras. We expected that adding the position will increase the model performance.

We trained two types of observation models with DT:

1. $OM_{o}$: We used all data to train the OM and the the same data to test the model (*o* denotes “optimistic”). This is an over-fitted model and we assumed it should provide the best performance for the given system model and sensor data. Although this model is not realistic, it gives us information about the capabilities of the approach under optimal conditions.
2. $OM_{p}$: We used the first run for training and the rest to test the model (*p* denotes “pessimistic”). We chose the first run as it is the only one containing all actions. This observation model gives us information about the performance of the system model in the case of very fuzzy observations.

#### 4.4.2. Hidden Markov Model

**Figure 9:**
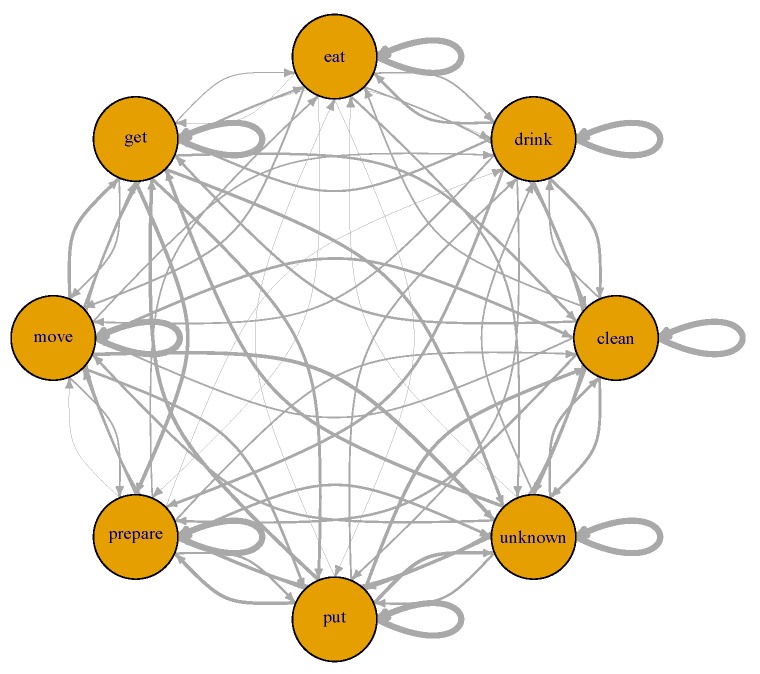
The HMM used for activity recognition. Each state represents an action class. Thicker lines indicate higher transition probabilities.

To compare the CCBM approach to the state of the art, we also built a hidden Markov model (HMM) both for activity and goal recognition. The number of hidden states in the HMM equals the number of action classes. Figure 9 shows a graphical representation of the modelled HMM.

The HMM has a transition matrix as well as prior probabilities for each state. We empirically estimated the transition matrix from the annotation of the training data by counting the number of state transitions. The state prior probabilities are based on the number of times a state appears in the training data. Two different transition models were examined:

- estimating the transition model from the data of all runs (general HMM, which we call $HMM_{g}$); and
- estimating the transition model separately for each run (specialised HMM, which we call $HMM_{s}$).

For the HMMs, we used the same two observation models as with the CCBM model.

##### Goal Recognition with HMM

The HMM was used for goal recognition, as proposed in [16]. This means an HMM was built for each possible goal. Each HMM was trained using all runs where this goal occurs. These HMMs were combined by introducing a common start state. The transition probabilities from the start state to the states of the sub-models were the normalised priors of the sub-model states. Note that the sub-models can have different number of states, when not all action classes occur in a run. This type of HMM is known as joint HMM [53]. This way, each state in the joint HMM is a tuple (action class, goal) and the probability of a goal is computed by marginalising over all states with the same goal.

### 4.5. Evaluation Procedure

To select the best combination of sensors for behaviour recognition and to evaluate the models’ performance, the following experiments were conducted:

1. Feature selection of the best combination of sensors: This experiment was performed to select the sensors that at most contribute to the model performance. The selected combination was later used when comparing the CCBM and HMM models.
2. Activity recognition of the action classes was tested with DT, HMM, and CCBM models.
3. Goal recognition of the types of meals and drinks as well as preparation of healthy/unhealthy meal/drink was studied.

#### 4.5.1. Feature Selection

We used the HMM for activity recognition to find the best feature combination, where by “feature” we mean the type of sensor. We did two types of evaluation: We first performed activity recognition with all feature combinations without the features from the depth cameras ($2^{12}=4096$ combinations). This feature selection was performed with $OM_{p}$ as the observation model and DT as classifier. $OM_{p}$ was chosen because it gives the most realistic assessment of the performance on new data ($OM_{o}$ is over-fitted and selecting features with this observation model would result in an over-fitted feature combination, i.e., in too many features).

This procedure results in accuracies for all feature combinations. We computed the accuracy using

(16) $$\mathrm{Accuracy}=\frac{\sum_{C}\lambda_{C}}{N}.$$

where *C* represents the action class; *N* represents all instances that are classified; and λ is the number of correctly recognised instances in *C*.

As we wanted to decide which features to use for the model evaluation, for each feature *f*, we calculated how much it contributes to the model performance.

1. We started by comparing the mean performance for the different feature combinations.
2. We decided that *f* may produce noise when the performance of the models which contain *f* is below the accuracy of the rest of the models;
3. For the model comparison, chose the feature combination with the highest accuracy.

We performed a similar procedure for all features combinations including the features from the depth cameras (($2^{15}=32,768$ combinations)). We performed this second evaluation separately as the first run (D1) does not contain the features from the depth cameras but it is the only dataset that contains all action classes. For that reason, we performed a leave-one-out cross validation. D1 was thus removed from the evaluation. For each feature combination, we performed 14 training runs: training with all of the runs except *i*, and evaluating the performance (accuracy) on run *i*. Then, the accuracy was calculated as the mean accuracy of the 14 runs.

#### 4.5.2. Activity Recognition

We used factorial design for both activity and goal recognition. For the activity recognition, three types of factors were examined:

1. **Algorithm:** This factor considered the different approaches to be compared (DT, HMM, and CCBM). The decision tree was our baseline and it gave information about the performance when applying only the observation model. In that sense, we expected the HMM and CCBM models to perform better that the DT.
2. **Observation model:** (optimistic pessimistic);
3. **System model:** (general/specific). In the case of DT, we did not have different system models.

The different dimensions we considered resulted in 10 models.

#### 4.5.3. Goal Recognition

Each dataset was annotated with one or several goals. Two kinds of goal annotations exist:

- Meal goals: pasta, coffee, tea, salad, chicken, toast, juice, potato, rice, water, cookies, and ready meal
- Healthy/unhealthy goals: healthy drink, unhealthy drink, healthy meal, and unhealthy meal

The goals for a given run can be seen in Table 2.

CCBMs (and HMMs) normally cannot recognise multiple goals. Instead, they recognise one goal for each time step. Ideally, they converge after some time, only recognising a single goal after this time. To perform multi-goal recognition (as required by this scenario), we examined two strategies for deriving multiple goals.

**Multiple goals strategy:** We collected all goals that were recognised at least once by the algorithm, and compared them with the set of true goals. The performance of each dataset was estimated by Formula (17).

(17) $$\mathrm{performance}\left(i\right)=\frac{|{\mathrm{est}}_{i}\cap {\mathrm{truth}}_{i}|}{|{\mathrm{est}}_{i}|}$$

where ${\mathrm{est}}_{i}$ is the set of recognised goals for dataset *i*, and ${\mathrm{truth}}_{i}$ is the set of true goals for dataset *i*. The overall performance was the mean of each datasets’ performance.

**“Pooled” goals strategy:** Each distinct set of goals was subsumed as a goal set. We performed goal recognition with this goal set. The goal of a run is said to be recognised correctly if the goal estimation of this run converged to the correct goal.

Furthermore, we used different types of goal priors:

1. **Uniform priors (uninformed):** In this case, all priors (i.e., $x_{0}$) have the same probability.
2. **Informed priors:** Here, the correct goal has two times the likelihood than the rest of the goals.

When applying factorial design to the goal recognition, the activity recognition factors do not apply, as each sub-HMM has been trained by using the data of all runs where the corresponding goal occurs. For that reason, for the goal recognition, we examined the following factors:

1. **algorithm** (HMM/CCBM);
2. **goal target** (Meal/Healthy);
3. **type of multigoal recognition** (multiple goals/single, “pooled” goals); and
4. **prior** (informed/uninformed).

This resulted in 16 models.

## 5. Results

### 5.1. Feature Selection without the Depth Camera Features

**Figure 10:**
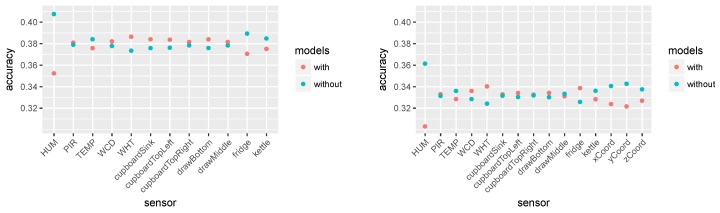
Mean accuracy with and without a given feature: (**Left**) the accuracy for all feature combinations without the camera features and using the first run (D1) for training and the rest for testing; and (**Right**) the accuracy of all feature combinations including the camera features and using leave-one-out cross validation.

In total, 4096 combinations of sensors were evaluated and from them the best combination was selected for the further experimentation. Using the procedure described in the previous section, the mean accuracy of the model with and without a given feature was calculated. Figure 10 (left) shows the results.

It can be seen that the humidity sensor reduces the performance of the model the most. The fridge electricity consumption, movement sensor, and temperature sensor also slightly reduce the mean performance. Interestingly, the fridge electricity consumption and movement sensors are both in the set of best performing feature combination (see Table 7). This indicates that taking the mean accuracy is probably not the best evaluation metric as some of the sensor combinations can reduce the usefulness of a sensor that otherwise brings relevant information to the model.

Table 7 shows the 10 worst and the 10 best combinations.

The best performing set of features is *fridge*, *cupboard top right*, *movement sensor*, *hot water consumption*, and *cold water consumption* with accuracy of 0.4332. This combination was later used in the remainder of the experiments.

### 5.2. Feature Selection with Locational Data from Depth Cameras

During the experiment, the position of the person estimated through depth cameras was also recorded. One hypothesis was that adding the position would increase the model performance. This hypothesis was not confirmed by the empirical results we obtained after adding the position to the existing sensor data. Below, we discuss the procedure for the data processing and the empirical results.

To address privacy concerns about RGB-D images being stored within the SPHERE system, there was a requirement for all features to be extracted at run time [45]. To obtain the positional and tracking information of any scene elements of interest, the SPHERE system makes use of a Kernelised Correlation Filter (KCF), augmented with Depth Scaling (DS-KCF) [54]. This is an extremely lightweight object tracker, which is capable of handling the complicated environments encountered by SPHERE, while running at around 180 frames per second.

By using DS-KCF to obtain a 2D bounding box for an individual in frame along with the depth information from the image, a 3D bounding box could be established to get the 3D position for each person within shot. This information was stored as Cartesian coordinates, and during data processing was handled in the same way as the rest of the sensor data (see Section 4.2). Three new columns were added for the x, y, and z position of the person extracted from the video data. To evaluate whether the video data provide any additional information that increases the model performance, we performed the feature selection process for all features including the depth camera features as presented in Section 4.5.1.

The 10 best and worst features are shown in Table 8.

It can be seen that the video data does not appear in the 10 best feature combinations. For this evaluation, the best feature combination is *kettle electricity consumption*, *cupboard top left*, *drawer bottom*, *temperature*, and *movement*. This feature combination does not include any camera features. When visually inspecting the video data compared to the actions from the ground truth, it became apparent that the camera data did not allow any conclusions about the currently performed activity. This could be interpreted in two ways. The performed activities were not related to the position of the person in the kitchen (e.g., the action “prepare” can take place at different locations within the kitchen). The second interpretation is that the position extracted from the camera was noisy and thus did not provide useful information for the model.

Figure 10 (right) shows the mean accuracy of all feature combinations with and without each feature. This shows how much each feature contributed to the overall performance. It shows that the camera features reduced the performance. Interestingly, in contrast to the results in Figure 10 (left), here the movement sensor and the fridge electricity consumption improved the performance. These two sensors were also used in the set of sensors selected for our experiments.

### 5.3. Activity Recognition

**Figure 11:**
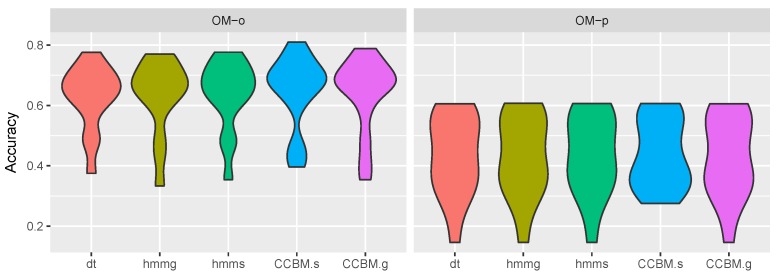
Activity Recognition results. *OM-o* refers to the optimistic observation model, *OM-p* to the pessimistic observation model, *dt* is decision tree, *hmmg* is the general HMM, *hmms* is the specialised HMM, *CCBM.s* is the specialised CCBM, and *CCBM.g* is the general CCBM.

Figure 11 shows the results for activity recognition. It depicts the distribution of the results for a given model over all runs. The accuracy was quite similar for all algorithms. There was a slight improvement in the recognition when using $CCBM_{g}$ with $OM_{o}$ compared to the HMM and DT models. In combination with $OM_{p}$, however, there was no difference in the performance of $CCBM_{g}$, $HMM_{g}$, $HMM_{s}$, and DT. Applying $CCBM_{s}$, there was improvement in the performance both when combined with $OM_{o}$ and $OM_{p}$. This improvement, however, was not significant compared to the rest of the models (0.038 < *p* value < 0.187, 81 < *V* < 86 when applying Wilcoxon signed rank test with N=15. *p* value of 0.038 was observed between $HMM_{g}$ and $CCBM_{s}$. Although it was slightly under the threshold value of 0.05, we considered that it was a borderline result, and not a significant difference between the two models).

The results show that the CCBM models did not significantly improve the performance for activity recognition and that the observation model had the largest effect on the accuracy. This was to be expected with regard to $CCBM_{g}$ as the model is very general (with 21 million valid plans), which allows coping with the behaviour variability but at the same time provides multiple explanations for the observed behaviour when the observations are ambiguous. Surprisingly, $CCBM_{s}$ also did not show significantly better performance. This could be partially explained with the fact that the ambiguous observations did not provide one-to-one mapping with the actions (i.e., multiple actions can have the same observation).

### 5.4. Goal Recognition

Next, we compared the performance of the informed CCBM model (we call it $CCBM_{i}$) and the uninformed CCBM mode ($CCBM_{u}$), with the informed HMM ($HMM_{i}$) and the uninformed HMM ($HMM_{u}$).

#### 5.4.1. Multigoal Model

**Figure 12:**
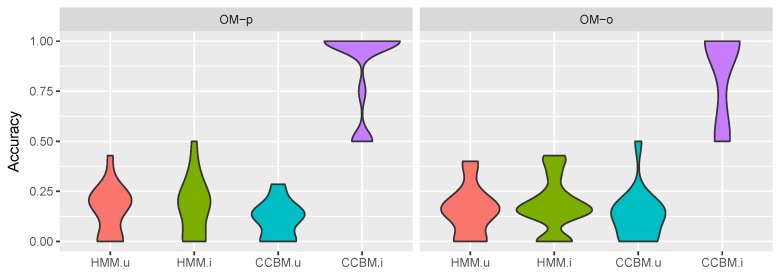
Multigoal recognition results, meal goals. *OM-o* refers to the optimistic observation model, *OM-p* to the pessimistic observation model, *HMM.u* is the HMM with uninformed a priori goal probabilities, *HMM.i* is the HMM with informed a priori goal probabilities, *CCBM.u* is the CCBM with uninformed a priori goal probabilities, and *CCBM.i* is the CCBM with informed a priori goal probabilities.

The results for goal recognition with multiple goals for the type of meal being prepared is depicted in Figure 12. It can be seen that $CCBM_{i}$ always performed significantly better than the rest of the models with a median accuracy of 1. We used Wilcoxon signed rank test to evaluate whether there was significant difference between the models’ performance. The results show that $CCBM_{i}$ combined with both $OM_{o}$ and $OM_{p}$ had a *p* value of under 0.0006 (V=120, Wilcoxon signed rank test with N=15), indicating that using CCBM model and informed priors indeed improved the goal recognition.

In difference to $CCBM_{i}$, $HMM_{i}$ performed comparable to $CCBM_{u}$ and $HMM_{u}$ and there was no significant improvement when using informed priors. This stands to show that CCBM models are able to provide significantly better goal recognition than the state-of-the-art HMM.

**Figure 13:**
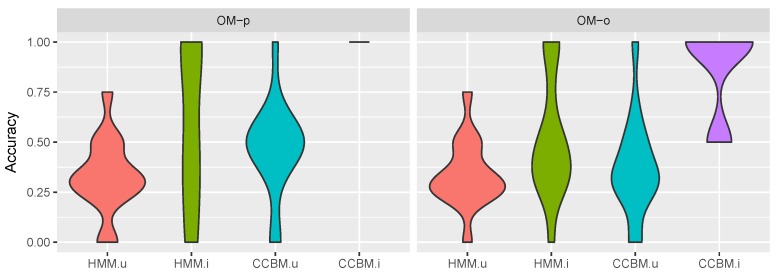
Multi-goal recognition results, healthy/unhealthy goals. *OM-o* refers to the optimistic observation model, *OM-p* to the pessimistic observation model, *HMM.u* is the HMM with uninformed a priori goal probabilities, *HMM.i* is the HMM with informed a priori goal probabilities, *CCBM.u* is the CCBM with uninformed a priori goal probabilities, and *CCBM.i* is the CCBM with informed a priori goal probabilities.

Figure 13 shows the results for recognising whether the meal or drink being prepared is healthy.

Once again $CCBM_{i}$, when combined with $OM_{p}$, performed significantly better than the rest of the models (*p* value <0.01; V=120,45,105 for $HMM_{u}$, $HMM_{i}$ and $CCBM_{u}$, respectively, using Wilcoxon signed rank test with N=15). Interestingly, $CCBM_{i}$ combined with $OM_{p}$ led to significantly better accuracy than when combined with $OM_{o}$. This was the opposite of what we expected, as the pessimistic observation model had an inferior activity recognition performance. This could be interpreted as a result of the very ambiguous observations, which allowed the CCBM model to rely on its symbolic structure and the assigned priors in order to recognise the goal. Although the priors played an important goal, it can be seen that $CCBM_{u}$ combined with $OM_{p}$ also performed better than when combined with $OM_{o}$. It also performed significantly better than $HMM_{u}$ (*p* value of 0.005, V=55) and better than $HMM_{i}$, which stands to show that, in the case of ambiguous observations, CCBM models are able to provide additional information that improves the goal recognition performance.

#### 5.4.2. “Pooled” Goals

**Figure 14:**
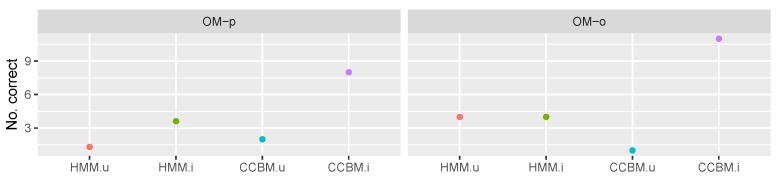
Single-goal recognition results, meal goals. *OM-o* refers to the optimistic observation model, *OM-p* to the pessimistic observation model, *HMM.u* is the HMM with uninformed a priori goal probabilities, *HMM.i* is the HMM with informed a priori goal probabilities, *CCBM.u* is the CCBM with uninformed a priori goal probabilities, and *CCBM.i* is the CCBM with informed a priori goal probabilities.

The results for the single goal recognition are depicted in Figure 14. It shows the number of goals recognised by the model (15 being the highest number achievable). In contrast to the multi-goal recognition, now both HMM models had a better performance than $CCBM_{u}$ (when combined with $OM_{o}$).

In combination with $OM_{p}$, $CCBM_{u}$ performed slightly better than $HMM_{u}$ but it was unable to outperform $HMM_{i}$. However, $CCBM_{i}$ still yielded the best results. In combination with $OM_{o}$, it was able to recognise 11 out of the 15 goals. However, the results show that, when using “pooled-goals” the performance of the CCBM model strongly depends on the observation model and the assigned priors. This differs from the results obtained when using the multiple goals strategy, where the models combined with $OM_{p}$ showed better performance.

## 6. Discussion

The results show that the CCBM modelling approach could reason about the actions and goals of the protagonist in real world cooking scenario based on noisy and ambiguous sensors. This stands to show that CSSMs are applicable to real world behaviour recognition problems in cases where the protagonist is acting in a goal-oriented manner. On the one hand, the combination of symbolic rule-based structure and probabilistic reasoning makes the approach attractive in situations where purely rule-based systems will have problems with the sensors’ ambiguity.

On the other hand, in difference to discriminative probabilistic approaches, the symbolic structure of CCBM allows integrating contextual information and causal dependencies that provide a mechanism for reasoning about the person’s goals and reasons behind the observed behaviour. This was also observed in the empirical results for goal recognition, which showed that the model is able to reason about the protagonist’s goals even in the case of very ambiguous observations (when using $OM_{p}$).

In difference to goal recognition, however, the results show that the model was seriously influenced by the observation model when performing activity recognition. This could be explained by the fact that the model is very general and has a high degree of freedom in selecting the next action. Combined with ambiguous sensors, the model is able to provide various explanations to the observations, which reduces the model performance. This problem can be addressed by either providing better action selection heuristics, or by tailoring the model in order to reduce the degree of freedom. In that respect, reinforcement learning methods could potentially be applied to learn more typical execution sequences.

**Figure 15:**
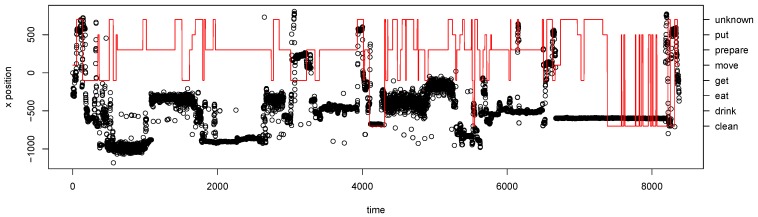
Example of the relationship between camera data and performed activity. The x-axis position extracted from the depth camera is given with black circles, while the annotated actions are given with red solid line.

Another interesting result is that the positional data from the depth cameras do not improve the model performance. This could have two causes: either there was a problem with the data, or the model did not make use of the location information. The latter assumption is reinforced when visually examining the location data (see Figure 15).

In the example, it can be seen that there is no obvious relation between the annotation and the location. This problem can be addressed by introducing additional locational information into the CCBM model (e.g., specific actions can be executed at a certain location in the kitchen).

Finally, the manual modelling of symbolic models is a time consuming and error prone process [55,56]. Additionally, it is often the case that the system designers are not the domain experts. That is especially true in medical applications, such as health monitoring. One potential solution to this problem is to automatically generate the CCBM models from textual sources provided by domain experts [57,58].

## 7. Conclusions and Future Work

In this work we investigated the practical application of CSSMs to the problem of AR and GR in a real world setting. To evaluate the approach, we used a sensor dataset containing the preparation of various meals and drinks. The results show that the approach performed AR with accuracy comparable to that of state of the art approaches such as DT and HMM. In the case of goal recognition, the approach was able to infer the prepared meal and its “healthy” status even when the AR performance was poor. As a conclusion, the approach showed the potential to infer one’s activities and goals that could hint at medical conditions or the progression of such even in unscripted scenarios and home settings.

In the future, we plan to explore several different directions. We intend to build a more fine-grained model that contains additional contextual details such as the concrete objects that are manipulated and the locational information. This will provide additional information about the person’s situation beside the goal being followed. It could also potentially improve the model performance when combined with the locational data from the depth cameras.

The model we built in this work had a very large state space (>400,000 states). A more fine-grained model will result in an even larger state space. To address this problem, we intend to investigate a lifted inference approach, which provides a mechanism for efficient reasoning in cases of observation equivalent states [59].

In this work, we analysed the behaviour of a single person preparing a meal. There is already evidence that CSSMs are also able to reason about multiple users in scripted experiments [33], thus we intend to investigate CSSMs for this purpose in future work.

We also intend to investigate combining deep learning methods with our approach in order to increase the model performance.

Finally, we plan to record a new dataset that contains the cooking activities of people with health-related conditions. We will use this dataset to validate the ability of our model to reason about the nutrition-related risks and conditions of patients in home settings. 

## Figures and Tables

**Table 1:** Existing CSSMs applied to activity and goal recognition problems.

Approach	Plan Rec.	Durations	Action Sel.	Probability	Noise	Latent Infinity	Simulation	Multiple Goals	Unscripted Scenario
[31]	■	□	■	■	□	■	no	□	□
[9]	■	□	■	■	□	■	yes	■	□
[33]	□	■	■	■	■	■	no	□	□
[12]	■	■	■	■	■	■	no	□	□
[11]	■	■	■	■	■	■	no	□	□
[34]	■	□	■	■	□	■	yes	□	□
[10]	■	□	■	■	■	■	yes	□	□
[35]	■	□	■	■	□	■	yes	■	□
[36]	■	□	■	■	□	■	yes	■	□

□ feature not included; ■ feature included

**Table 2:** Types of meal and length of execution sequence in a dataset. “Number of Actions” gives the discrete actions required to describe the sequence (i.e., it gives the number of actions executed during the task). “Time” gives the duration of the recording in time steps. Time steps were calculated using a sliding window over the data, which was originally in milliseconds (see Section 4.2). “Meal” gives the eventual result of the food preparation.

Dataset	# Actions	Time	Meal
D1	153	6502	pasta (healthy), coffee (unhealthy), tea (healthy)
D2	13	602	pasta (healthy)
D3	18	259	salad (healthy)
D4	112	3348	chicken (healthy)
D5	45	549	toast (unhealthy), coffee (unhealthy)
D6	8	48	juice (healthy)
D7	56	805	toast (unhealthy)
D8	21	1105	potato (healthy)
D9	29	700	rice (healthy)
D10	61	613	toast (unhealthy), water (healthy), tea (healthy)
D11	85	4398	cookies (unhealthy)
D12	199	3084	ready meal (unhealthy), pasta (healthy)
D13	21	865	pasta (healthy)
D14	40	1754	salad (healthy)
D15	72	1247	pasta (healthy)

**Table 3:** The actions schema for the ontology.

1) (move<location><location>)	5) (eat<meal>)
2) (get<item><meal>)	6) (drink<meal>)
3) (put<item><meal>)	7) (clean)
4) (prepare<meal>)	8) (unknown)

**Table 4:** Object sets in the ontology.

**Meal**	chicken, coffee, cookies, juice, pasta, potato, readymeal, rice, salad, snack, tea, toast, water, other
**Item**	ingredients, tools
**Location**	kitchen, study

**Table 5:** Excerpt of the annotation for run D1. Time here is given in milliseconds.

Time	Label
1	(unknown)
3401	(move study kitchen)
7601	(unknown)
10,401	(prepare coffee)
31,101	(unknown)
34,901	(clean)
47,301	(unknown)
52,001	(get tools pasta)
68,001	(get ingredients pasta)
86,301	(prepare pasta)
202,751	(get tools pasta)
221,851	(get ingredients pasta)
228,001	(prepare pasta)

**Table 6:** Parameters for the different models.

Parameters	${\mathrm{CCBM}}_{g}$	${\mathrm{CCBM}}_{s}$
Action classes	8	8
Ground actions	92	10–28
States	450,144	40–1288
Valid plans	21,889 393	162–15,689

**Table 7:** Accuracies for the 10 worst and 10 best sensor combinations without the camera features.

**10 Worst Combinations**	
**Features**	**Accuracy**
fridge, drawer middle, drawer bottom, humidity, movement	0.2688
fridge, drawer middle, drawer bottom, humidity, movement, water cold	0.2691
fridge, drawer bottom, humidity, movement, water cold	0.2692
fridge, drawer bottom, humidity, movement	0.2692
fridge, cupboard top left, humidity, movement	0.2694
fridge, cupboard top left, drawer middle, humidity, movement	0.2694
fridge, humidity, movement, water cold	0.2695
fridge, drawer middle, humidity, movement, water cold	0.2695
fridge, cupboard sink, humidity, movement, water cold	0.2695
fridge, draw middle, humidity, movement	0.2695
**10 Best Combinations**	
**Features**	**Accuracy**
drawer bottom, cupboard sink, water hot, water cold	0.4307
drawer middle, drawer bottom, water hot, water cold	0.4308
cupboard top left, drawer middle, drawer bottom, water hot, water cold	0.4308
drawer middle, drawer bottom, cupboard top right, water hot, water cold	0.4308
fridge, drawer bottom, movement, water hot, water cold	0.4325
fridge, movement, water hot, water cold	0.4330
fridge, cupboard top left, movement, water hot, water cold	0.4330
fridge, draw middle, movement, water hot, water cold	0.4330
fridge, cupboard sink, movement, water hot, water cold	0.4330
fridge, cupboard top right, movement, water hot, water cold	0.4332

**Table 8:** Accuracies for the 10 worst and 10 best sensor combinations with the camera features.

**10 Worst Combinations**	
**Features**	**Accuracy**
fridge, cupboard top left, drawer bottom, cupboard top right, humidity, xCoord	0.2199
fridge, cupboard top left, drawer bottom, cupboard sink, humidity, xCoord	0.2199
fridge, cupboard top left, drawer middle, cupboard sink, humidity, movement, xCoord	0.2194
fridge, cupboard top left, drawer middle, humidity, movement, xCoord	0.2189
fridge, cupboard top left, cupboard sink, humidity, movement, xCoord	0.2170
fridge, cupboard top left, drawer middle, cupboard top right, cupboard sink, humidity, xCoord	0.2167
fridge, cupboard top left, drawer middle, cupboard top right, humidity, xCoord	0.2162
fridge, cupboard top left, drawer middle, cupboard sink, humidity, xCoord	0.2162
fridge, cupboard top left, drawer middle, humidity, xCoord	0.2158
fridge, cupboard top left, cupboard top right, cupboard sink, humidity, xCoord	0.2149
**10 Best Combinations**	
**Features**	**Accuracy**
kettle, cupboard top left, drawer bottom, temperature, movement	0.4911
kettle, cupboard top left, drawer bottom, cupboard top right, temperature, movement	0.4911
kettle, cupboard top left, drawer bottom, cupboard sink, temperature, movement	0.4911
kettle, cupboard top left, drawer bottom, cupboard top right, cupboard sink, temperature, movement	0.4911
kettle, cupboard top left, cupboard sink, temperature, movement	0.4902
kettle, cupboard top left, cupboard top right, cupboard sink, temperature, movement	0.4901
kettle, cupboard top left, drawer middle, drawer bottom, cupboard sink, temperature, movement	0.4901
kettle, cupboard top left, drawer middle, drawer bottom, cupboard top right, cupboard sink, temperature, movement	0.4901
kettle, drawer bottom, cupboard sink, temperature, movement	0.4892
kettle, drawer bottom, cupboard top right, cupboard sink, temperature, movement	0.4892

## Abbreviations

The following abbreviations are used in this manuscript:

AR	Activity Recognition
CCBM	Computational Causal Behaviour Model
CSSM	Computational State Space Model
DS-KCF	Depth Scaling Kernelised Correlation Filter
DT	Decision Tree
ELAN	Multimedia Annotator
GR	Goal Recognition
HMM	Hidden Markov Model
JSON	JavaScript Object Notation
KCF	Kernelised Correlation Filter
OM	Observation Model
PDDL	Planning Domain Definition Language
PR	Plan Recognition
SPHERE	Sensor Platform for HEalthcare in a Residential Environment
